# Virus-induced brain pathology and the neuroinflammation-inflammation continuum: the neurochemists view

**DOI:** 10.1007/s00702-023-02723-5

**Published:** 2024-01-23

**Authors:** Jeswinder Sian-Hulsmann, Peter Riederer

**Affiliations:** 1 Department of Human Anatomy and Medical Physiology, University of Nairobi, P.O. Box 30197, Nairobi, 00100 Kenya; 2 University Hospital Wuerzburg, Clinic and Policlinic for Psychiatry, Psychosomatics and Psychotherapy Margarete-Höppel-Platz 1, 97080 Würzburg, Germany; 3 Department of Psychiatry, University of Southern Denmark, Winslows Vey 18, 5000 Odense, J.B Denmark

**Keywords:** Neurodegeneration, Parkinson’s disease, Alzheimer’s disease, Viruses, Microglia and neuroinflammation, Inflammation, Cytotoxicity, Immunology

## Abstract

Fascinatingly, an abundance of recent studies has subscribed to the importance of cytotoxic immune mechanisms that appear to increase the risk/trigger for many progressive neurodegenerative disorders, including Parkinson’s disease (PD), Alzheimer’s disease (AD), amyotrophic lateral sclerosis, and multiple sclerosis. Events associated with the neuroinflammatory cascades, such as ageing, immunologic dysfunction, and eventually disruption of the blood–brain barrier and the “cytokine storm”, appear to be orchestrated mainly through the activation of microglial cells and communication with the neurons. The inflammatory processes prompt cellular protein dyshomeostasis. Parkinson’s and Alzheimer’s disease share a common feature marked by characteristic pathological hallmarks of abnormal neuronal protein accumulation. These Lewy bodies contain misfolded α-synuclein aggregates in PD or in the case of AD, they are Aβ deposits and tau-containing neurofibrillary tangles. Subsequently, these abnormal protein aggregates further elicit neurotoxic processes and events which contribute to the onset of neurodegeneration and to its progression including aggravation of neuroinflammation. However, there is a caveat for exclusively linking neuroinflammation with neurodegeneration, since it’s highly unlikely that immune dysregulation is the only factor that contributes to the manifestation of many of these neurodegenerative disorders. It is unquestionably a complex interaction with other factors such as genetics, age, and environment. This endorses the “multiple hit hypothesis”. Consequently, if the host has a genetic susceptibility coupled to an age-related weakened immune system, this makes them more susceptible to the virus/bacteria-related infection. This may trigger the onset of chronic cytotoxic neuroinflammatory processes leading to protein dyshomeostasis and accumulation, and finally, these events lead to neuronal destruction. Here, we differentiate “neuroinflammation” and “inflammation” with regard to the involvement of the blood–brain barrier, which seems to be intact in the case of neuroinflammation but defect in the case of inflammation. There is a neuroinflammation-inflammation continuum with regard to virus-induced brain affection. Therefore, we propose a staging of this process, which might be further developed by adding blood- and CSF parameters, their stage-dependent composition and stage-dependent severeness grade. If so, this might be suitable to optimise therapeutic strategies to fight brain neuroinflammation in its beginning and avoid inflammation at all.

## Introduction

Parkinson’s (PD) and Alzheimer’s (AD) are progressive neurodegenerative diseases with unknown aetiology. Nevertheless, a plethora of evidence generated from studies suggests the close involvement of risk factors such as ageing, genetics, and pathogen-induced neuroinflammation. Viral infections in the central nervous system (CNS) are significantly more frequent compared to other pathogens, including bacteria, fungi, and protozoa (Romero and Newland 2003). Strikingly, a large study of 450,000 people (above the age of 60 years and of European ancestry) from Finland and the UK found that viral infections (mainly neurotropic viruses) increased the risk of development of neurodegenerative disorders, including PD and AD (Levine et al. 2023). The viruses reported by this study included influenza virus, herpes zoster virus, enteroviruses, Epstein–Barr virus (EBV), and Herpes simplex virus.

Incidentally, the acute consequences related to virus infections are associated with a heightened host’s inflammatory response rather than the viral load itself (Coates et al. 2017). Thus, neuroinflammation resulting from virus (chiefly neurotropic by nature) exposure appears to be strongly associated with neurological sequelae despite viral clearance and may play an instrumental role in the early stages of disease development. As such, a plethora of evidence indicates neurodegeneration may be a casualty of virus-invoked inflammation and disruption of cellular protein metabolism (Leblanc and Vorberg 2022; Smeyne et al. 2021).

## Putative pathways and mechanisms for viral-associated neurodegeneration

### Neuroinflammation

Viruses access the CNS via two major routes: the blood supply (mumps and measles viruses (Griffin 2014)), and some migrate through the peripheral nerves. Rabies virus and poliovirus employ peripheral motor neurons to enter the CNS (Jackson 2014). It has been reported that viral-infected mononuclear cells can gain access to the CNS within 12 h after infection with coxsackie virus B3 (Tabor-Godwin et al. 2010).

The blood–brain barrier (BBB) is a close-fitting vascular barrier composed of brain microvascular endothelial cells, astrocytes, and pericytes (Ben-Zvi et al. 2014). It tightly regulates the entry of components from the blood respectively peripheral nervous system into the CNS (Rausch et al. 1983). The BBB restricts the entry of toxins, macromolecules, and trespassing pathogens. However, some regions of the CNS, like the circumventricular organs, may allow the entry of pathogens since they do not have complete protection from the BBB (Daneman and Prat 2015). Alternatively, BBB itself may be “leaky” or dysfunctional, allowing the free passage of toxins, pathogens, immune cells, and their products. This would also irregulate ions and signal homeostasis (Profaci et al. 2020), which may have debilitating effects on the physiological functions of neurons and represent a prelude to neurodegeneration to ensue.

**Fig. 1:**
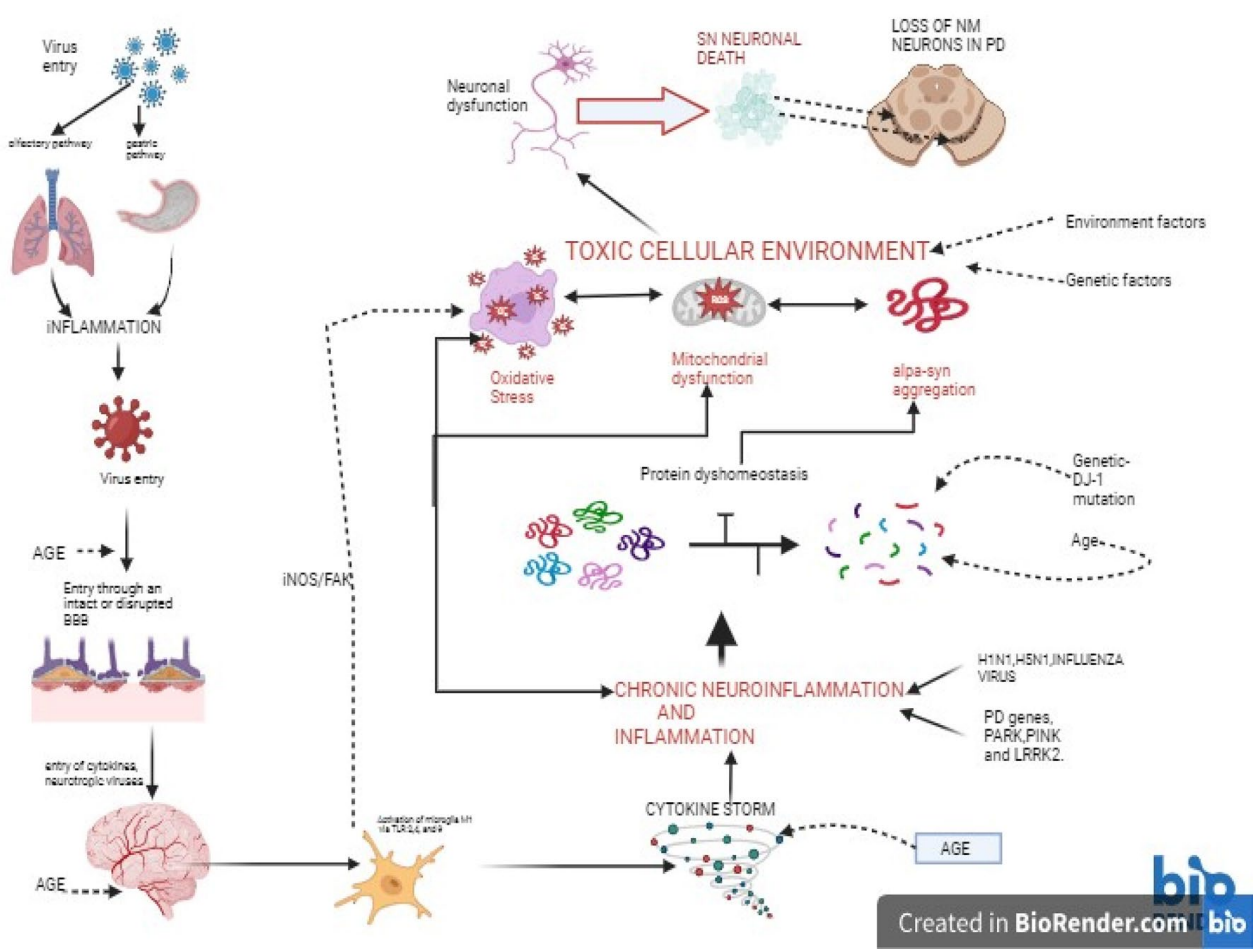
Sequence of events that may be related to viral-related infection coupled with other risk factors in Parkinson’s disease. Viruses (HIN1, H5N1, FLAVI, HEP.C, HIV, etc) gain entry through the olfactory or/and gastric avenues, inciting neuroinflammation in the respiratory and gastrointestinal tract respectively. This leads to the release of proinflammatory cytokines and chemokines, which can disrupt the integrity of the blood-brain barrier (BBB) by producing fenestrations on its endothelial cells. Thus, making it “leaky” and allowing the passage of pathogens/toxins to the brain. On gaining entry to the brain, these cytokines can activate microglia (M1 cells) via toll-like receptors (2,4, and 9), which generates further production of pro-inflammatory cytokines (IL-1, IL-6, and TNF-α, etc.) and onset neuroinflammation in the brain. Also, chronic neuroinflammation has been implicated in familial PD. Neuroinflammation may contribute to the dysregulation of protein clearance pathways, resulting in the build-up of misfolded α-synuclein, mitochondrial malfunction, and cytotoxic reactions resp. oxidative/nitrative stress by the generation of reactive free radicals. Eventually, these neurotoxic processes coupled with other risk factors (age, genetic predisposition, and environmental factors) ultimately destroy the dopaminergic nigral neurons in PD. Key: BBB-blood brain barrier; PD-Parkinson’s disease; SN substantia nigra; NM neuromelanin; iNOS-inducible nitric oxide/FAK pathway;TLR-Toll-like receptors; IL-Interleukin;TNF- α tumor necrosis factor; α-syn-alpha synuclein; HSV-1 herpes simplex virus -1;Hep.C hepatitis C virus; Flavi-flaviviruses; H1N1 Influenza A virus; H5N1 avian influenza; and HIV human immunodeficiency virus

Virus or viral-related consequences may be instrumental in impairing the integrity of the BBB (Fig. 1). Inflammation generated by systemic viral infection may be a potential candidate in the damage of the BBB, thereby permitting viral neuroinvasion (Eugenin 2006; Chen et al. 2018).

Increasing evidence supports the involvement of neuroinflammatory mechanisms as key players in commanding viral-related neurotoxicity (Pathak and Sriram 2023). Following virus-associated infections, neuroinflammation may manifest in various areas in the CNS, such as encephalitis in the brain, meningitis (meninges), and/or myelitis (spinal cord) (Tansey and Goldberg 2010; Klein et al. 2017). Alternatively, the inflammation can occur concurrently in different areas (meningoencephalitis). Interestingly, although viral pathology exerts a significant role in inducing malfunction of the CNS physiological processes and mechanisms, the immune response against the virus can also contribute to dysfunction and disease. Thus, neuroinflammatory cells and mediators may serve as a “Trojan Horse” (Figs. 1, 3) once they gain their illegal entry into the brain, biding their time before invoking chaos and cellular destruction.

Depending on the virus, they can replicate within neurons, glia, and microglia. Of the three glial cells, only microglia and astrocytes execute a cytotoxic effect. The oligodendrocytes like the neuronal cells are the casualties of the destructive inflammatory processes (McGeer and McGeer 2007).

**Fig. 2:**
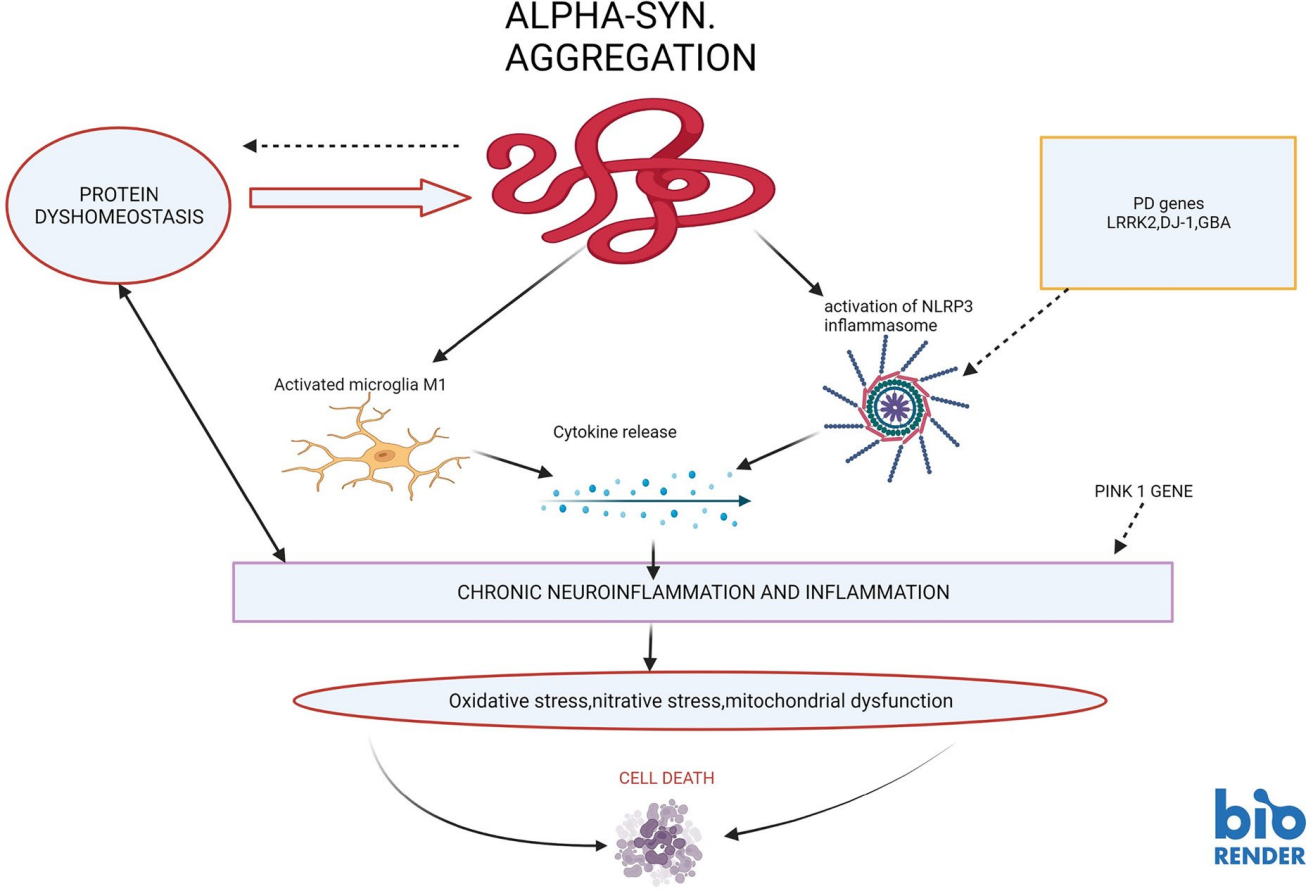
Consequences of α-synuclein aggregation in PD. Accumulates of misfolded α-synuclein can provoke an array of consequences that exacerbate the burden of neuronal destruction. Including; activation of microglia, further disruption of protein metabolism, and activation of NLRP3 inflammasome (there also may be involvement of genetic components). Subsequently, these processes marshal in a “cytokine storm” or excess release of cytokines, thereby ushering in a state of chronic neuroinflammation and neuronal death

Microglia can serve as a double-edged sword, and in that in response to neuronal damage or an invading pathogen, it can execute both a protective and destructive action (Li and Barres 2018). This dual role probably relates to the two distinct microglial phenotypes, M1 and M2 (Prinz and Jung 2019). M1 exerts pro-inflammatory and neuro-destructive activities (Figs. 2, 5), whereas M2 microglia express markers such as (cluster of differentiation, CD) CD163, and CD206 that reduce the rate of inflammation and tissue repair (Orihuela et al. 2015).

Microglial cells are key regulators of inflammation related to neurodegenerative disorders. Neuronal cell destruction can activate glia/microglia and trigger neuroinflammation via the release of inflammatory mediators and reactive oxygen species (Wolf et al. 2017). Free radicals can initiate cytotoxic processes such as oxidative stress thereby further adding to the neuronal cell death toll. Also, activated microglial can produce pro-inflammatory cytokines and chemokines to suppress and eradicate the viral infection (Amarante-Mendes et al. 2018). Usually, the second wave of cytokine releases (post-infection) promotes viral clearance (Guo and Thomas 2017). However, if this release goes unchecked coupled with dysregulation of anti-inflammatory reactions, a “cytokine storm” follows an aggressive hyperinflammatory response. This can have direct and catastrophic consequences by precipitating serious immune pathology including acute respiratory distress syndrome, coagulopathy, extensive tissue destruction, and multi-organ failure resulting in death (Verbist and Nichols 2019; Zanza et al. 2022). Microgliosis also generates pro-inflammatory cytokines including, (interleukin, IL) IL-1β, IL-6, and Tumor necrosis factor-α (TNF-α), which could curtail further damage to the CNS, however, they also have the potential to be destructive to the neurons (Smith et al. 2012). Interestingly, biological features (Fig. 1) of the host such as age, gender, and obesity may affect the pathological magnitude of the cytokine storms (Fajgenbaum 2020). In addition, it was observed in a study in Jakarta that all the patients infected with the SARS-CoV-2 virus (COVID-19) that experienced a cytokine storm in the pulmonary tissue suffered from comorbidities such as hypertension and diabetes (Ramatillah et al. 2022; Gu et al. 2021). Thus, the health status of the host plays a key role in the vulnerability to the immunopathology of cytokine storms.

Astrocytes can also release both pro and anti-inflammatory molecules and studies have shown that inflammatory cytokines such as IL-1β and TNF-α can induce astrocytes to release chemokines (CXCL10, CCL1, and CXCL13, Szpakowski et al. 2022). Thus, cytokines released from the microglia can activate astrocytes. In addition, astrocytes can modulate microglial activity. Raised levels of cytokines have been reported in serum, cerebrospinal fluid, and postmortem brain areas in PD patients (Blum-Degen et al. 1995; Mogi et al. 1994a, b; 1995a, b; 1996a, b; Wijeyekoon et al. 2020). This in conjunction with evidence for reactive microgliosis and astrocytes in the substantia nigra (SN) of PD patients (McGeer et al. 1988; McGeer and McGeer 2004, 2007,2008; Hunot and Hirsch 2003) and AD (McGeer et al. 1988; Eikelenboom et al. 1994; McGeer et al., 2006), reflects an activated inflammatory situation and supports the involvement of neuroinflammation in the pathogenesis of the disease. (Hirsch and Standaert 2021).

Under physiological conditions, microglia are neuroprotective and vital in maintaining neural connections. However, microgliosis may contribute to destructive inflammatory processes in the pathological state (such as PD or AD). More importantly, the SN may be particularly susceptible to the activity of the microglia since there is a fivefold greater number of these cells in this area compared to the cortex or hippocampus as found in the rat model (Kim et al. 2000). Thus, it has been suggested that microglial activation in the SN exerts a more neurotoxic state as opposed to a protective function. The damage executed by activated microglia includes the release of pro- and anti-inflammatory agents and more importantly, cellular toxins such as superoxide radicals. Thus, in the diseased state, the microglia exert a menacing role by triggering free radical-mediated oxidative stress resulting in dopaminergic cell loss in SN and overall neurodegeneration (Simpson and Oliver 2020). Many in vitro studies and animal models of PD have demonstrated the destructive effects of activated microglia (Joers et al. 2017). In addition, a recent study reported that modulation of reactive microglia cells with cerebral dopamine neurotropic factor supported fetal mesencephalic graft survival by attenuating the immune responses in a hemi-parkinsonian rat model (Tseng et al. 2022).

Toll-like receptors (TLRs) are expressed chiefly on microglia and play a key role in stimulating inflammatory responses. Indeed, microglial cells are involved in neuroinflammatory processes in response to invading pathogens (viruses/bacteria) via TLRs. Interestingly, TLRs increase macrophage motility mediated via the inducible nitric oxide synthase (iNOS) /FAK pathway (Maa et al. 2011). The iNOS has been shown to exacerbate oxidative and nitrative stress (Fig. 1) which leads to neuroinflammation and loss of neuronal function in the mouse prion model (Bourgognon et al. 2021). In addition, it has been suggested that interleukin IFN-γ pairs with TLRs in the microglia release nitric oxide which in turn produce neuronal malfunctioning (Schilling et al. 2021). These findings support the involvement and key role that neuroinflammation may play in neurodegenerative disorders’ pathogenesis. Interestingly, overexpression of TLRs has been documented in brain tissue and blood cells in PD (Drouin-Ouellet et al. 2014). The upregulation of the TLRs is associated with the aggravation of neuroinflammation. Thus, this led to the notion that TLRs (particularly, TLR2, TLR4, and TLR9) mediated neuroinflammation may exert a dominant neuro-destructive force that can contribute to the casualty of nigral dopaminergic cells loss in PD (Heidari et al. 2022). An overall chronic state of neuroinflammation, coupled with activation of microglial cells and hypercytokinemia can have grave consequences and in the case of PD (Fig. 2), it can prompt the characteristic destruction of nigral neurons (Tansey and Goldberg 2010).

**Fig. 3:**
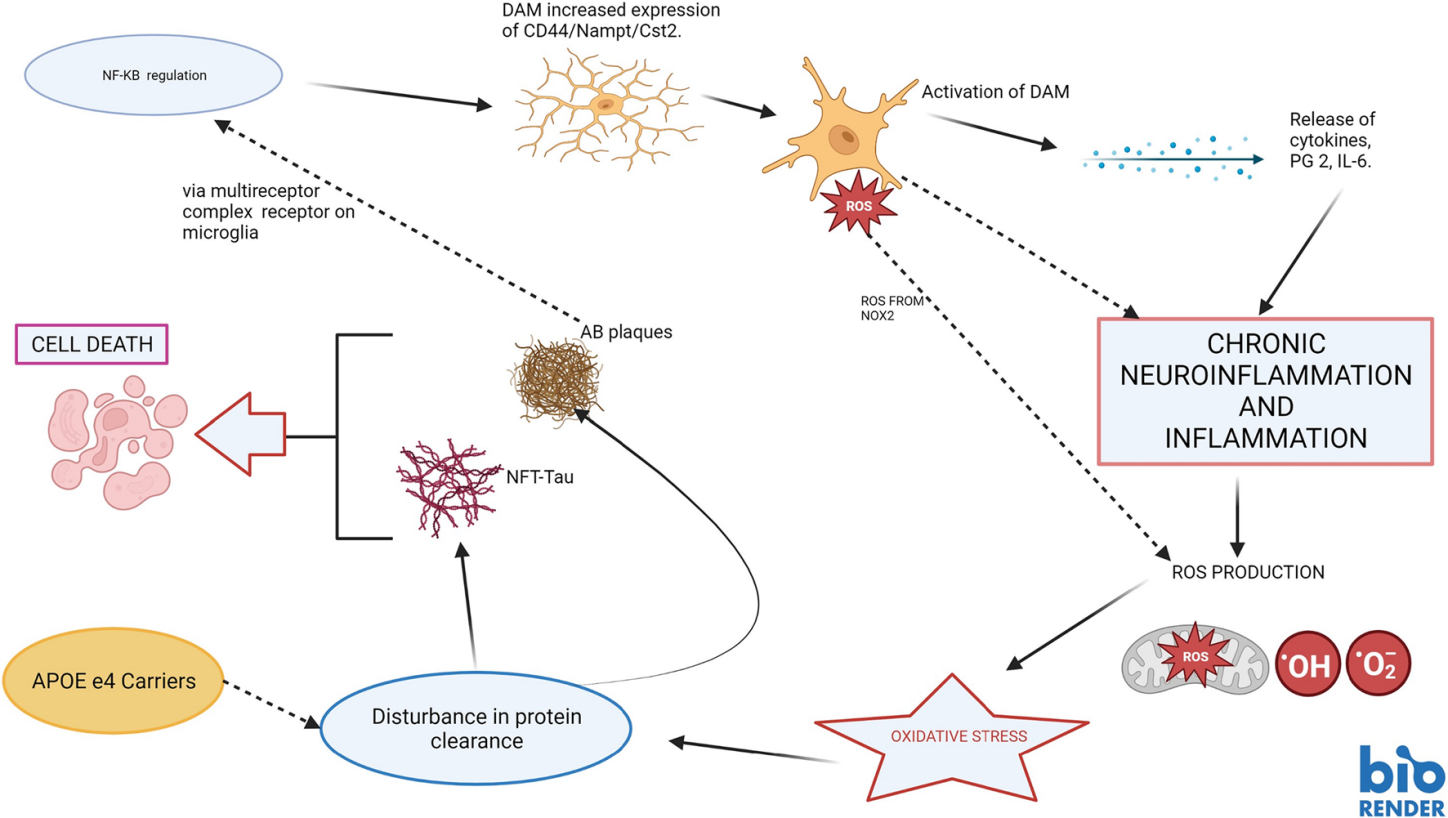
Neuroinflammatory-mediated degenerative mechanisms that may be operating in the pathogenesis of Alzheimer’s disease, NF-κB regulates the enhanced expression of pro-inflammatory DAM genes (Cd44, Nampt, and Cst2). An increase in the expression of these genes can prompt the activation of DAM causing the generation of inflammatory mediators (for example, PG E2, IL-6) and inflammation. Neuroinflammation can elicit cytotoxic processes such as oxidative stress, and disturbance of protein clearance pathways, thereby resulting in a build-up of Aβ deposits and tau neurofibrillary tangles. The presence of these pathological markers herald’s neurodegeneration characteristics of the disease. Key: NF-κB nuclear factor kappa B; ROS reactive oxygen species; DAM disease-associated microglia; PG E2 prostaglandin E2; IL-6-Interleukin 6; NOX2 NADPH OXIDASE 2; NFT-tau neurofibrillary tangles containing tau and Aβ amyloid beta

The gathering of the activated microglia in brain areas exhibiting marked neuronal loss confirms the occurrence of destructive neuroinflammation in AD (Hansen et al. 2017). Thus, similar to PD, neuroinflammation generated by microglia has also been implicated in the pathophysiology of AD (McGeer et al. 1988; 2006; Mandreka-Colucci and Landreth 2010; Eikelenboom et al. 1994), although the two disorders differ greatly in symptoms, pathological markers, and areas demonstrating neurodegeneration. Genetic studies suggest that microglial cells play a major role in the development of the disease and also express risk factors for AD in the brain. The presence of the AD pathological hallmarks amyloid β (Aβ) deposits/ plaques in close proximity to the microglia (Hansen et al. 2017) offers an opportunity for some possible interaction (s). Indeed, activation and replication of microglial cells close to Aβ plaques is a characteristic siting in AD. Acute microglial inflammation may be initiated by exposure to Aβ aggregates (Baik et al. 2019). Interestingly, pathomechanisms associated with AD (Fig. 3) may be linked to disease-associated microglia (DAM) (Keren-Shaul et al. 2017). The pro-inflammatory DAM genes*, CD44, Nampt, and Cst2* are implicated in AD (Rangaraju et al. 2018). NF-κB (nuclear factor kappa B) regulates the high expression of *CD44, and CD45,* which in turn leads to the activation of the proinflammatory DAM. In vitro studies suggest that the association of neurofibrillary Aβ with microglia leads to the activation of these cells. This accounts for the powerful inflammatory reaction that is initiated in response to the deposition of Aβ plaques in the brain (Fig. 3). It has been proposed that the fibrillar forms of Aβ engage with a multicomponent receptor complex on the microglia (Bamberger et al. 2003). This interaction stimulates the NF-κB mediated phenotypic microglial activation coupled with amplification of free radical production and cytokine expression. This receptor complex has been reported to play a chief role in the development of AD (Combs et al. 2001). Incidentally, activated microglia cells may also aggravate another pathological feature in AD, tau pathology (Ayyubova 2022).

Furthermore, as discussed earlier the activation of microglia can orchestrate a host of processes such as neuroinflammation, production of cytokines, and cytotoxic events elicited by reactive oxygen and nitrogen species, which can lead to the ultimate destruction of neurons (Hickman et al. 2018). In addition, the activated microglia may facilitate a chronic neuroinflammatory milieu that causes neuronal death directly or by stimulation of astrocytic-mediated neurotoxic processes (astrogliosis; Pekny and Pekna 2016) that ultimately lead to degeneration.

However, there seems to be a continuous development of neuroinflammation to profound inflammation. There is no evidence so far that neuroinflammation is detectable in the brain of young healthy individuals. However, it can be detected in brain tissue of aged people (Chung et al. 2009; DiSabato et al. 2016). Indeed, endogenous toxic metabolites like cytokines, chemokines, oxidative stress parameters etc., generated by the physiological processes of neuronal aging with loss of neurons seem to facilitate low grade activation of microglia, the resident innate immune cells. At this stage, the BBB blocks the transport of peripheral immune cells into brain tissue and there is not much antigen presentation to activate T-cells (Streit et al. 2004; Tohidpour et al. 2017). Of notion, acute and/or chronic infection, traumatic brain injury or exogenous toxic compounds may disturb the BBB and enter the brain so that peripheral immune cells, macrophages, T-cells, B-cells, and other circulating blood components pass compromised BBB and further facilitate neuronal and glial disturbances leading to massive expression of cytokines resp. chemokine levels and expressing enhanced major histocompatibility complex molecules (MHC). In addition, activated astrocytes may release endogenous compounds, inclusive growth factors (DiSabato et al. 2016; Streit et al. 2004). Depending on the type of infectious agents, viruses, bacteria, environmental toxins and their potency to compromise the integrity of the BBB and to enter certain brain areas of preference, they may or may not trigger an inflammatory response (Tohidpour et al. 2017; Porcher et al. 2021). As such, and as the terminus technicus “neuroinflammation” has not been clearly defined and often is used to be synonymous with “inflammation, Aguzzi et al. (2013) have contributed to the definition of “neuroinflammation”. Here, we enlarge this concept and propose a staging as given in Table 1:

**Table 1:** Is there a neuroinflammation-inflammation continuum?

1: The young (healthy) brain: no neuroinflammation
2: The aged brain: neuroinflammation with intact BBB but endogenous inflammatory processes in the absence of infectious diseases (Chung et al. 2009; DiSabato et al. 2016)
3: The neuroinflammed brain: infectious agents; chronic neuroinflammation but intact BBB (Yacoubian et al. 2023)
4: The inflammed brain: infectious agents; opening of the BBB, entry of macrophages, T-cells, B-cells, proteins etc. into brain areas causing inflammation (Mestre et al. 2021)

Impairment of the immune system affects both the innate and adaptive responses (Iwasaki 2012; Hill et al. 2022) thus contributing to the susceptibility for a variety of disorders of the aged individual with activated microglia expressing high levels of (Major histocompatibility, MHC) MHC-II and co-stimulatory antigens (Andronie-Cioara et al. 2023) like in PD and AD (McGeer and McGeer 1988).

### Modulation of microglia M1 and M2

As discussed earlier, the microglial cells appear to orchestrate a key role in the neurodegenerative processes. It is vital therefore to highlight the receptors /cytokines/factors/ signal pathways that may alter the microenvironment involved in the modulation of microglia from M2 to M1 to curtail cellular destruction (Yao and Zu 2020). The secretion of interleukin IL-4 or IL-13 from T-helper cells 2, favors the polarization of microglia to the M2 phenotype. In turn, the M2 microglia release anti-inflammatory cytokines to diminish inflammatory reactions thereby supporting the repair of injured cells. Whereas, cellular injury and the related release of pro-inflammatory cytokines TNF-α and interferon-gamma (IFN-γ), results in the microglia polarization to the M1phenotype. Thus, it is imperative to regulate the activation of the microglia not only to maintain homeostasis in the brain but also to avert or dilute the neuro-destructive pathways (Song et al. 2016).

Indeed, an imbalance of microglial polarization, with a dominance of the M1 phenotype and malfunction of the M2 neuroprotective type, fosters the occurrence of AD (Yao and Zu 2020). Furthermore, Aβ has been reported to activate microglia inflammation via the toll receptor 2 (TLR2) (Jana et al. 2008). Similarly, activation of TLR2 has been suggested to contribute to α-synuclein aggregation (via the autophagy/lysosomal pathways) in PD (Dzamko et al. 2016). Collectively, this endorses the role of TLR2 in the modulation of microglial polarization. In addition, Toll receptor 4 is activated by lipopolysaccharide (present in Gram-negative bacterial membrane) resulting in the polarization of M1 microglia (Ciesielska et al. 2020).

Other receptors/molecules/ factors/signal pathways involved in the polarization of microglial include (Fig. 5):

- Receptor-mediated routes for microglial modulation. For instance, the peroxisome proliferator receptor is a chief regulator of M2 phenotype and can therefore attenuate inflammatory responses (McTigue 2008).
- Neuropeptide Y has been shown to significantly hinder microglial activation by blocking the lipopolysaccharide-induced Fc-receptor-mediated phagocytosis (Ferreira et al. 2011). Therefore, neuropeptide Y may play a role in the management of microglial phagocytic activity. In vivo experiments showed that glial cell line-derived neurotrophic factor inhibited the polarization of M1 (Rocha et al. 2012).
- A transcription factor, micro RNA-124 has been suggested to manage the activity of microglia via down-regulating the expression of cytosine-cytosine-adenosine-adenosine-thymidine-enhancer-binding protein-α. This notion was demonstrated by the maintenance of the microglia in a quiescent state after the administration of micro RNA-124 in hyperalgesia induced in rats (Willeman et al. 2012).
- The interleukin family of cytokines is an important signaling molecule in the immune processes (Fields et al 2019). A shift of M1 to M2 phenotype polarization is supported by anti-inflammatory cytokines including: insulin-like growth factor (Labandeira-Garcia et al 2017), IL-4 (Tang et al. 2019), IL-13 (Colonna and Butovsky 2017) and IL-33 (Fu et al. 2016).
- The JAK/STAT transducer signaling pathway exerts a vital role in inflammatory reactions (Ruganzu et al 2021). STAT1 and STAT3 support the polarization of M1 microglia and consequently the release of pro-inflammatory cytokines (Lan et al 2017). In contrast, IL-4 activates STAT6, which in turn induces the appearance of M2 phenotype (Lan et al. 2017).
- Free radicals generated from cellular oxidative stress have the propensity to shift the expression of microglia from M2 to M1 (Ren et al 2020). Unregulated release of these cytotoxic species by NADPH oxidase may precipitate inflammation-related pathology. This concept is demonstrated by the raised expression of NADPH oxidase in the microglia in AD (Choi et al. 2011).
- NF-κB is a principal transcription factor involved in the activation of the M1 microglia cells (Zhang et al. 2013). Kawai and co-worker (2007) reported that inhibition of NF-κB by modulators produced microglial polarization from M1 to M2 type.
- Transforming growth factor-β-activated kinase 1 is a key regulator for inflammation and cell viability via activation of NF-κB and MAPK (Mihaly et al 2014). In addition, MAPK advocates M2 microglial polarization, which augments its anti-inflammatory actions (Zhang et al. 2019a).

Nevertheless, suppression of the M1 microglial phenotype may not be sufficient to halt inflammatory-mediated neurodegenerative pathways (Zhang et al. 2017), M2 polarization may also be required to attain full therapeutic potency (Du et al. 2018).

## Disruption of neuronal protein accumulation

### Parkinson’s disease

The presence of misfolded α-synuclein containing Lewy bodies in the remaining surviving nigral dopaminergic neurons represents the pathological hallmark of PD (Gibb et al. 1991). Interestingly, a mouse lentiviral model was used to mediate the selective accumulation of α-synuclein in the microglia, which produced the progressive loss of dopaminergic cells (Bido et al. 2021). The inability of the protein homeostasis pathways to cope with excessive α-synuclein aggregation in the microglia resulted in a “phagocytic exhaustion state” and a cellular toxic environment (perhaps due to the production of free radical species), which caused dopaminergic neuronal destruction. These findings offer a number of plausible pathomechanisms in PD, including malfunction of α-synuclein homeostasis, a build-up of misfolded α-synuclein, and microglial-mediated or associated cytotoxic neuroinflammation (Fig. 2). The role of environmental triggers resp. pathogens to modify the structure of α-synuclein has recently been reviewed in detail (Riederer et al. 2019; Sian-Hulsmann and Riederer, 2021; Linard et al. 2022; Huynh et al. 2023). In addition, α-synuclein accumulation can stimulate the microglia M-1-like polarization and impairs the microglia protein regulation mechanisms (phagocytosis and autophagy) and activation of NLRP3 inflammasomes (Lv et al. 2023). NLRP3 can also be activated by DAM to start the processing of caspase-1 and release of pro-inflammatory cytokines, IL-1β, and IL-8 (Blevins et al. 2022; Pike et al. 2022). This serves as the premise of targeting NLRP3 inflammasome to block chronic inflammation in many diseases. In addition, genes implicated in PD including, LRRK2, DJ1, and GBA, also regulate these processes, thereby highlighting the role of disturbed protein metabolism in the pathogenesis of the disease (Lv et al. 2023).

Microglial cells serve an important role in the homeostasis of the α-synuclein in the brain via autophagy and phagocytosis. Autophagy via microglia also blocks the NLRP3 inflammasomes invoked neuroinflammation (Lv et al. 2023). Consequently, under pathological conditions, DAM and polarized M-1 microglia may accentuate the accumulation of the aggregated α-synuclein via damage-associated proteins (DAMP), although some studies suggest a neuroprotective role for DAM (Deczkowska 2018). This supports an association between the appearance of α-synuclein inclusions and the dysregulation of protein metabolism in PD. DAM may support a chronic inflammation and oxidative stress state by generating reactive free radical species. Indeed, free radicals can be generated from NADPH oxidase 2 (NOX2) in DAM, and activated NOX2 is related to inflammation (Fig. 3), DAMP signaling, and β-amyloid plaque deposition (Simpson and Oliver 2020). Interestingly, the transcription factor Nrf2 stimulates the expression of antioxidant NADPH dehydrogenase 1 in microglia. This vital finding can be employed in the advocation of cellular protective therapy from inflammation and related oxidative stress associated with neurodegeneration (Hickman et al. 2018).

The second mechanism of protein regulation is autophagy. Synucleinophagy is a selective autophagy that is mediated by microglia for clearance of neuron-released α-synuclein. Thus, microglial α-synuclein degradative pathway appears to exert a vital role in neuroprotection. This notion is demonstrated by the enhanced accumulation of incorrectly folded α-synuclein coupled with the destruction of nigral dopaminergic neurons, due to disturbances inflicted on microglial autophagy in mice expressing human α-synuclein (Choi et al. 2020). Experiments using knock-down DJ-1 genes in microglia showed an impaired autophagic uptake and clearance of α-synuclein and dysfunction of autophagy-dependent of p62 and LC3 proteins (Nash et al. 2017). Indeed, the synucleinophagy clearance of misfolded α-synuclein is mediated via TLR4-NF-κB-p62 (Choi et al. 2020). The cellular protective role of DJ-1 against oxidative stress is demonstrated by enhanced vulnerability to the neurotoxicity of dopamine cells in DJ-1 deficiency in microglia (Ariga et al. 2013). Furthermore, DJ-1 mutations ascribe to the autosomal recessive form of PD *park 7* (Repici and Giorgini 2019). Although out of all the causative genes (PARKIN, PINK-1) related to familial PD, DJ-1 mutations only account for a small number. In addition, the presence of the oxidized form of DJ-1 in the brains of PD patients betrays the operation of cytotoxic oxidative stress. These findings support the concept of the involvement of some underlying genetic default that ascribes/contributes to the overall build-up of misfolded α-synuclein in PD.

Therefore, although microgliosis does not appear to directly initiate α-synuclein aggregation, it can evidently contribute to the overall total load of misfolded protein generally. Indeed, PD animal experimental models demonstrate and endorse a principal role of activated microglia and NLRP3 inflammasome in both α-synuclein neuropathology and dopaminergic neuronal loss (Pike et al. 2022). Incidentally in experimental studies, dopamine has been shown to halt the activation of human microglia NLRP3 inflammasome (Pike et al. 2022). This may account for the vulnerability of the depleted nigral dopamine neurons to the onslaught of cytotoxic neuroinflammatory processes in PD (Fig. 2).

Analogous to PD, the involvement of aggregation of rogue proteins is also a characteristic pathological feature in AD. The presence of abnormal or misfolded Aβ peptide deposits and neurofibrillary tangles (NFTs) containing tau protein in the brain is a characteristic and well-documented histopathological observation in AD (Alzheimer 1907 resp. Alzheimer et al. 1995).

Generally, infections produced by particular pathogens such as viruses or bacteria *(helicobacter pylori*) or fungi (Malassezia) have been linked with an enhanced risk of the manifestation of PD (Wang et al. 2022; Limphaibool et al. 2019). A recent epidemiology study (Cannon and Gruenheid 2022), postulated the “multiple microbe” hypothesis and found that the risk of PD was greater in subjects that were seropositive to a number of microbes (viruses, bacteria, and fungi) compared to controls that were negative. It was suggested that the manifestation of PD-like symptoms due to these microbial infections is probably related to common pathophysiological processes employed including systemic neuroinflammation, accumulation of misfolded α-synuclein, and dysfunction of mitochondrial respiratory pathways. Also, in the cases of familial PD, the gene mutations may also influence the host-microbe interactions. Indeed, the familial PD genes (PARK2, LRRK2, and PINK 1) have been shown to cause misregulation of immune mechanisms (Figs. 1, 2) and thus genetically mediated modulation of immune consequences may exercise a more integral role in neurodegeneration (Patrick et al. 2019; Baizabal-Carvallo and Alonso-Juarez 2021).

The advent of **post-encephalitis parkinsonism (PEP)** was exhibited after about 10 years in around 50% or more of those that survived encephalitis lethargica produced by **H1N1 influenza virus** (von Economo’s disease) between 1915 and 1927 (Jankovic and Tan 2020; Foley 2009). Interestingly, it shares many features in common with the idiopathic form of PD including motor abnormalities, dystonia, and neuropathological changes such as degeneration of SN neurons (Davis et al. 2014; Jankovic 2022; Toledano and Davies 2016). However, the characteristic “pill-rolling” movement observed in PD is absent. In addition, there appears to be a difference in the time frame for pathological development in the case of PEP, the loss of dopamine neurons associated with the viral infection (Cocoros et al. 2021) and it shows slower progression compared to idiopathic PD (Jang et al. 2009a).

It has been postulated that two possible mechanisms are related to viral-related parkinsonism (Sulzer et al. 2020). The first one is para-infectious and produces almost immediate neuronal destruction coupled with parkinsonism, whereas the second mechanism is post-infectious and produces a slower neuronal cell death. The two mechanisms are not exclusive and can occur concomitantly. More importantly, both pathways of viral-induced cellular degeneration are mediated through neuroinflammation. Furthermore, there are some hypotheses offered related to viral-associated neurodegeneration (Sulzer et al. 2022). The findings related to PEP led to the evolvement of the “dual-hit” hypothesis to the development of PD (Hawkes et al. 2007). It suggests that a neurotropic pathogen (probably of viral origin) gains entry to the brain via two sites, the olfactory bulb and gastric mucosa via the vagus nerve, thus the name dual-hit hypothesis (Fig. 1). At these sites, the neurotropic pathogen triggers α-synuclein pathology (Braak et al. 2003). Subsequently, the culprit pathogen is associated with neuroinflammation leading to the destruction of the nigral cells. Alternatively, another virus-related neurodegeneration theory has been postulated, the “hit and run” hypothesis (Scarisbrick and Rodriguez 2003). This suggests that the virus invasion can initiate a powerful inflammatory response that can elicit cytotoxic neuronal destruction which can still operate even after viral load has been cleared (Hara et al. 2021). The “hit and run” hypothesis appears to suggest a very viable possibility for viral-related pathogenesis in neurodegeneration.

The neuropathology of PEP shows marked neuronal loss and gliosis throughout the brainstem with a focus on the SN. PEP is a tauopathy and NFTs have been demonstrated in nearly all brain areas of interest (Jellinger 2009; Hoffmann and Vilensky 2017). However, and in contrast to progressive supranuclear palsy (PSP) but similar to AD NFTs are composed of both 3R and 4R isoforms (Hoffmann and Vilensky 2017). Of note, LBs and Lewy neurites or any other α-synuclein deposits are absent in PEP (Jellinger 2009).

As human post mortem studies from PEP to identify viral particles so long after the pandemic are difficult and connected with many problems (see Cadar et al. 2021; McCall et al. 2001; Reid et al. 2001) more recent experimental work using **H5N1 influenza virus** gives way to understand, at least in part, the mechanisms underlying virus-induced pathology leading to neuropsychiatric disorders. Jang et al. (2009b) demonstrated that H5N1 spreads from the peripheral nervous system to the central nervous system. There the virus interacted with α-synuclein phosphorylation and aggregation, caused nigral degeneration with loss of dopaminergic neurons in the SNpc as well as activation of microglia and neuroinflammation (Jang et al. 2009b). Depending on the experimental conditions H5N1 infection in the long-term induces transient loss of the dopaminergic phenotype but causes long-lasting inflammatory response (Jang et al. 2012).

PEP following exposure to other viruses has been frequently reported in man and rodents (Jang et al. 2009a; Leta et al. 2022). The viruses include influenza A (Gamboa and Yahr 1973), flaviviruses such as West Nile Virus (Robinson et al. 2003; Sejvar 2014), Japanese B encephalitis, St Louis encephalitis, Hepatitis C (Wang et al. 2022), HIV retrovirus and SARS-CoV-2 RNA positive virus. Strikingly, infections with these viruses may result in the manifestation of some cardinal parkinsonian features such as tremor, cogwheel rigidity, and bradykinesia (Beatman et al. 2016) and pathology in the midbrain.

**Flaviviruses** proteins orchestrate the rearrangement of the endoplasmic reticulum membrane required to form replication complexes for the virus (Barrett and Weaver 2012; Sempere and Armando 2019; Thomas et al. 2014). Subsequently, flavivirus reproduction is accompanied by reactive oxygen species-induced endoplasmic reticulum oxidative stress and apoptotic destruction of virally infected cells (Zhang et al. 2019b). α-Synuclein expression was found to be increased in primary neurons infected with West Nile Virus (Beatman et al. 2016). Thus, native α-synuclein executes a neuroprotective role which is lost in the pathogenesis of PD. Indeed, it has been documented that deletion of the α-synuclein gene in mice leads to an increase in viral propagation in the brain coupled with a decrease in neuronal survival rate (Beatman et al. 2016). In contrast, no reports were found relating Japanese encephalitis virus and α-synucleinopathy, thereby suggesting a different mode of pathogenesis (Misra 2002). Although West Nile Virus and Japanese encephalitis virus as well as mosquito-borne alphavirus infect and cause vast cellular damage to the brain, particularly in the dopaminergic systems including the SN, basal ganglia, and other regions, cerebellum, and brain stem (Solomon 2004; Bantle et al. 2019), the Parkinsonian symptoms appear to be temporary and resolve over time. Another flavivirus, the St Louis encephalitis virus is closely related to the Japanese encephalitis virus. It also produces brain inflammation or encephalitis and consequent parkinsonism (Curren et al. 2018). Up to 20% of Dengue virus infections affect the brain with encephalitis, encephalopathy and movement disorders such as parkinsonism (Hopkins et al. 2022).

**Filovirus**, like Ebola virus infrequently demonstrate neurological signs, but when present they consist of meningitis, encephalopathy and seizures (Sagui et al. 2015). Ebola virus is suggested to bypass the BBB. Using quantitative MR-relaxometry, 18F-fluorodeoxyglucose PET and immunohistochemistry Schreiber-Stainthorp et al. (2021) in a monkey model describe BBB disruption with extravasation of fluid and albumin into extracellular space. The regional hypermetabolism corresponded to mild neuroinflammation, sporadic neuronal infection and apoptosis as well as increased glucose transporter-3 (GLUT-3) expression (Schreiber-Stainthorp et al. 2021).

Epidemiological evidence is indicative of an association between **Hepatitis C** infections and the manifestation of PD. Indeed, a Taiwanese study reported that a previous infection with the Hepatitis C virus increased the risk of subsequent development of PD (Odd ratio 1.39; Wu and Kang 2015). These finding were confirmed subsequently by Wijarnpreecha and colleagues (2018). The virus primarily causes neuroinflammation-mediated neuronal destruction. It enhances the release of pro-inflammatory cytokines, which leads to the onset of cytotoxic processes particularly in the dopaminergic areas of the brain. The predilection of the pathology in dopamine neurons probably ascribes the subsequent risk for the development of PD. Also, the virus is associated with the downregulation of the astrocyte-derived factor (this is neuroprotective), which exacerbates neuronal cell destruction (Wijarnpreecha et al. 2018).

Recent evidence has been provided to demonstrate a link between **Severe Acute Respiratory Syndrome Corona Virus-2 (SARS-CoV-2**) and PD. Coronavirus disease 2019 (COVID-19) affects the lower and upper respiratory tract with involvement of the peripheral and central nervous system (Harrison et al. 2020; Wu et al. 2023). As such the virus displays difficulties in breathing, cough, fever, headache, loss of taste and/or smell, fatigue and several neurological manifestations including acute cerebrovascular diseases, conscious disturbances, loss of cognitive abilities, attention disorder, anxiety, insomnia, depression and eventually problems of motor coordination as well as typical non-motor symptoms accompanying PD (Conte et al. 2021; Juan et al. 2021; Saniasiaya et al. 2019; Lopez-Leon et al. 2021; Premraj et al. 2022; Efsthathiou et al. 2022; Wang and Perez 2023). Therefore, the notion is of interest that human post mortem studies demonstrated neuropathological findings in several brain regions including the basal ganglia, thalamus, hippocampus cingulum and cortical areas (DaSilva-Torres et al. 2022).

TLRs, mainly TLR4 activation seem to cause neuroinflammation (Heidari et al. 2022). SARS-CoV-2 spike protein might bind to TLR4 and the interaction of SARS-CoV-2 and ɑ-synuclein has been discussed by Conte (2021). As ACE2-virus binding is low in the CNS Szabo et al. (2023) discuss the interaction of SARS-CoV-2 and TLR2. TLR2 is an innate immune receptor, which interacts with multiple viral components including the envelope protein of SARS-CoV-2 and plays an important role in PD and Alzheimer disease.

Szabo et al. (2023) hypothesize that TLR2 may play a critical role in the response to SARS-CoV-2 infiltration in the CNS.

In vitro studies demonstrate that SARS-CoV-2 proteins directly interact with ɑ-synuclein and cause Lewy-like pathology in the presence of ɑ-synuclein overexpression (Wu et al. 2022).

SARS-CoV-2 nucleocapsid (N) protein speeds ɑ-synuclein aggregation, while SARS-CoV-2 spike (S) protein has no effect on α-synuclein aggregation (Mesias et al. 2022), thus providing molecular insights into the impact of SARS-CoV-2 post-infection and the oligomerization of α -synuclein (Semerdzhiev et al. 2022).

SARS-CoV-2 infection in Macaques with mild-to-moderate disease in post mortem brain analyses showed activated microglia in the parenchyma as well as intracellular ɑ-synuclein aggregates (Philippens et al. 2022). In addition, SARS-CoV-2 infection of transgenic mice expressing human angiotensin converting enzyme 2 (ACE 2) drives NLRP3 inflammasome activation in human microglia (Albornoz et al. 2022). Combined experimental and computational methods show moderate binding between two SARS-CoV-2 proteins and ɑ-synuclein (Mesias et al. 2022). Altogether, such data suggest SARS-CoV-2 related neurological disorders, which should be confirmed by human post mortem studies (Riederer and Ter Meulen 2020). Indeed, Müller and Benedetto (2023) and Hill et al. (2022) discuss that ineffective immune response may be the reason that the virus is present for longer times, involves the vascular system, spreads within the brain and facilitates processes as that given above leading to chronic neuroinflammation with an increasing risk for the long-covid syndrome (NICE 2020; Müller and Riederer 2023) with psychiatric symptomology, depression (Benedetti 2023), fatigue and cognitive impairment (Penninx et al. 2021; Riederer and Ter Meulen 2020).

SARS-CoV-2 and its link to a-synuclein pathology opens the door to speculate about the potency of this virus to trigger parkinsonism. Wang et al. (2023a) conclude a two-year retrospective cohort study following COVID-19 infection is associated with a higher risk of developing neurodegenerative disease, that the risk of developing PD is increased for the first year after COVID-19 but that there is no further risk of PD after that time (Wang et al. 2023b). Indeed, the appearance of parkinsonism during or immediately after COVID-19 infection represents a VERY rare event (Cavallieri et al.^2022^; Akilli and Yosunkaya 2021). However, movement disorders started 13 days on average after the initial onset of COVID-19 and in a fraction of patients (22%) ataxia, myoclonus, tremor, parkinsonism was still present after a follow-up period of 75 ±3 weeks (Schneider et al. 2023).

To conclude this, it is known that SARS-CoV-2 affects neuronal populations relevant for the pathogenesis of PD, but there is no evidence that SARS-CoV-2 triggers neurodegeneration in the acute phase of infection and the early post-infection phase (Lingor et al. 2022; Szabo et al. 2022). This concurs with experimental data in which mice were administered a non-lesion dose of the parkinsonian toxin 1-methyl-4-phenyl-tetrahydropyridine (MPTP) infected with SARS- CoV-2 (Smeyne et al. 2022). There was no loss of dopaminergic neurons in the SNpc but a significant increase in microglial activation in the striatum and the SNpc (Smeyne et al. 2022). Of note, human post mortem studies from brain autopsies in COVID-19 do not show lesions of the nigrostriatal system (Mukerji and Solomon 2021; Goerttler et al. 2022; da Silva-Torres et al. 2022; Martin et al. 2022; Crunfli et al. 2022; Solomon et al. 2023), although viral RNA is still present several days after death (Skok et al. 2021). This indicates that there is no evidence of neurotropism for SARS-CoV-2 (Lebrun et al. 2023) although the outcome of the long-covid syndrome cannot be judged at this time (Davis et al. 2023). However, late-stage PD may be highly susceptible to critical COVID-19 infection and risky outcome (Zhai et al. 2021).

**MERS-CoV** infection induces a strong inflammatory response already in the acute phase (Mahallawi et al. 2018). In human DPP4 transgenic mice infected with MERS-CoV viral antigens were detected in brain areas four days post-infection with affection of neurons and astrocytes followed by a damage of the BBB, microglia activation and inflammatory cell infiltration, which may be caused by compliment activation (Jiang et al.^2021^).

**Human immunodeficiency virus (HIV)** type 1 transactivator of transcription (HIV-1Tat) protein can promote the activity of matrix metalloproteinases (MMPs) such as MMP-9 in astrocytes, which has the potential to damage the BBB and promotes the monocyte infiltration into the CNS (Sung et al. 2009; McArthur et al. 2003). This influx of monocytes symbolizes an early inflammatory event in HIV-associated encephalitis, dementia, and neurocognitive disorders (HAND) (Marino et al. 2020). In addition, mice injected with HIV-1 Tat lead to the activation of cyclooxygenase 2, which in turn subdued the expression of the tight junctions thereby encouraging the opening of the BBB (Pu et al. 2007).

HIV infection affected several million adults and children worldwide and it has been estimated that at least one third of the adults will develop a dementing illness (Janssen et al. 1992; McArthur et al. 1993; see also Koutsilieri et al. 2001) with a poor prognosis (Harrison et al. 2008). HIV-induced dementia with cognitive dysfunction and behavioral abnormalities is associated with motor disorders inclusive parkinsonism. Absence of a direct viral infection of neurons has been described (Takahashi and Yamada 1999) in contrast to glial cells (astrocytes, microglia; Wahl and Al-Harthi 2023) and it has been assumed that neurotoxins released by infected cells are causal for neurodegeneration leading to a dementia-parkinsonism syndrome (Glass and Johnson 1996). Excitotoxicity has been a focus of research to enrol the mode of action of the HIV dementia-parkinsonism syndrome (Koutsilieri et al. 2001). In fact, enhanced glutamatergic activity has been detected in patient suffering from AIDS as well as in HIV-positive patients without AIDS symptomology as summarised by Koutsilieri et al. (2001). Moreover, this group suggested dysregulation of the dopaminergic system as a pathogenic factor for NeuroAIDS (Koutsilieri et al. 2001) with a loss of dopamine in SIV-infected monkeys (Koutsilieri et al. 2002a, 2002b). Studies with the simian model of HIV as well as the chronically HIV-infected T-lymphoblasts ACH-2 demonstrated that dopamine induced a concentration dependent HIV activation (Scheller et al. 2000), a finding agreeing with those showing that dopaminergic substances such as L-DOPA and the monoamine oxidase inhibitor selegiline accelerate generation of vacuoles in the simian model of HIV infection (Czub et al. 1999, 2001, 2002b; Koutsilieri et al. 2002a) as is with metamphetamine in a HIV-cat model (Phillips et al. 2000). Indeed, dopamine is involved in the regulation of HIV gene expression in neuronal cells and cells of the immune system (Sawaya et al. 1996; Rohr et al. 1999). In contrast, memantine, a glutamatergic N-methyl-D-aspartate receptor antagonist, is beneficial in treating HIV infection in the simian model of HIV (Meisner et al. 2008). HIV-positive individuals show evidence for an activated brain innate immune response and increased brain inflammation associated with poorer cognitive performance (Vera et al. 2016; Wojna 2022) and depression (Mudra-Rakshasa-Loots et al. 2022). Of interest, experimental work in which supernatants of primary monocyte-derived macrophages (MDM) infected with HIV (HIV/MDMs) were used to treat primary cultures of rat oligodendrocyte precursor cells (OPC) during their differentiation to mature oligodendrocytes, HIV/MDMs inhibited OPC maturation (Roth et al. 2021). Additional studies showed that glutamate or AMPA- respective kainic acid receptor agonists phenocopied this effect, probably by inducing an excitotoxic stress response (Roth et al. 2021).

The notion is of interest that depending on the dose of the virus MRI studies of the brain demonstrate variation in the intensity of brain area lesions. For example, HIV shows predominantly cortical atrophy and basal ganglia lesions, EBV demonstrates striatal hyperintensity and basal ganglia necrosis, West Nile Virus (WNV) has a focus on bilateral basal ganglia, thalamus or pontine lesion, while Japanese Encephalitis Virus (JEV) shows bilateral thalamic involvement or substantia nigra lesion (Leta et al. 2022). PEP demonstrates widespread neurodegeneration in subcortical and brainstem areas (Itoh et al. 2000; Jellinger 2009). Such variations in the intensity of brain area lesion seem to have a clinical relevance insofar as the phenomenology shows differences with the consequence of levodopa treatment response to parkinsonian symptoms (Leta et al. 2022). In fact, HIV, EBV, WNV show poor levodopa response, while JEV PD symptoms can be treated successfully with levodopa and influenza virus demonstrates mild improvement (Leta et al. 2022).

As the pattern of virus-induced degenerative processes varies with regard to brain areas and especially striatum and substantia nigra we conclude that it is predominantly the nigro-striatal dopaminergic system that is involved in virus-induced neurodegeneration, focusing either the striatum (poor levodopa response (MSA-P type related?) or the substantia nigra (good levodopa response; PD type).

Of note, Theiler’s virus and other viruses such as neurotropic strains of mouse hepatitis virus readily and relative selectively infect the SN-tyrosine hydroxylase positive neurons following intranasal inoculation of mice (Oliver et al. 1997). Therefore, it is not farfetched to assume that the dopaminergic neurons of the SN play a primary role in the debilitating viral attack. This is, because in humans about 80–90% of SN neurons contain neuromelanin (NM; Hirsch et al. 1988, 2003). NM under physiological conditions binds neurotoxic substances/pathogens and eventually concentrate them but may release them under conditions of changes in the composition of the cytoplasma (Nagatsu et al. 2023; Riederer et al. 2021, 2023).

### Alzheimer’s disease

**Fig. 4:**
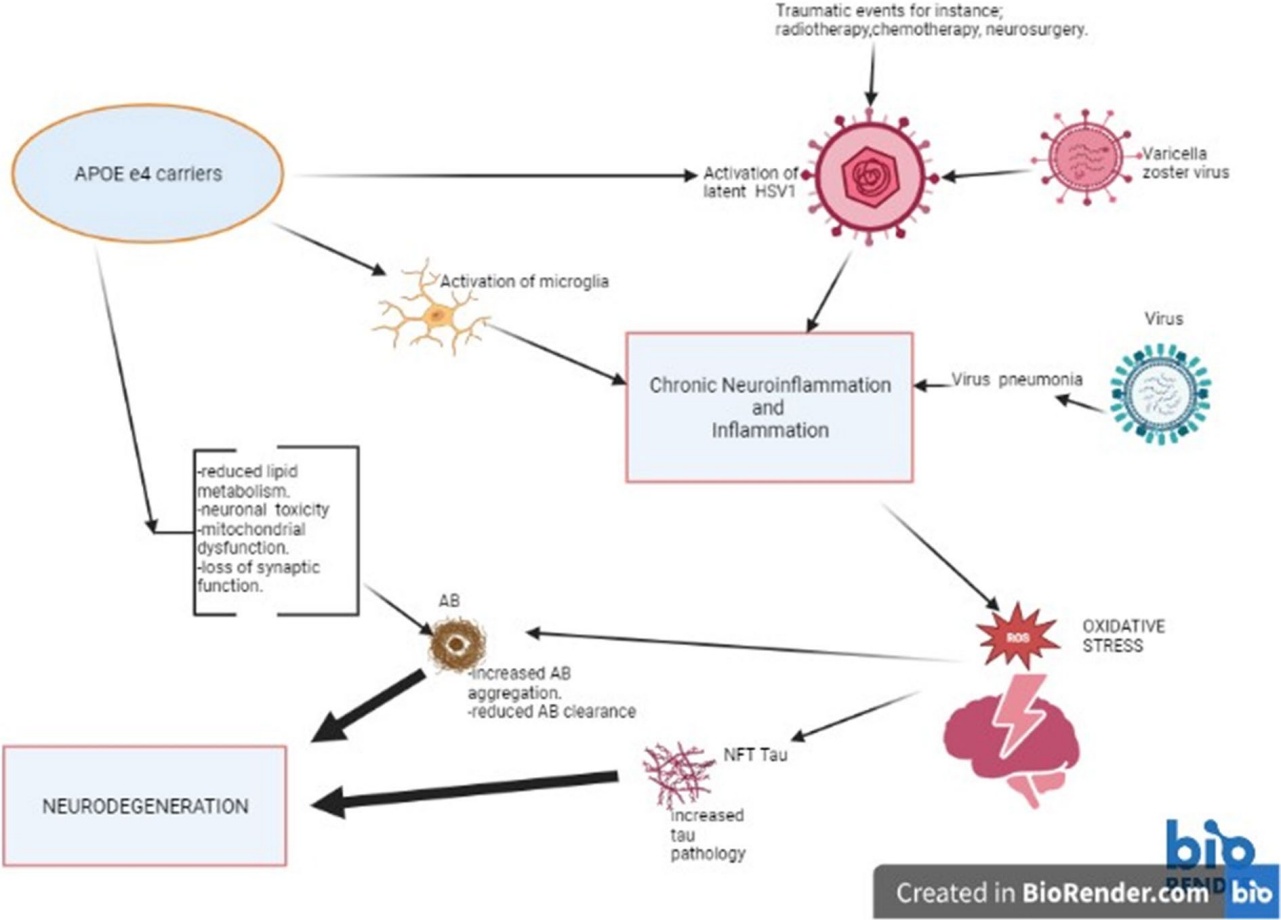
Apolipoprotein E e4 (ApoE e4) function in Alzheimer’s disease, Carriers of APOE e4 gene bestow the operation of many cellular toxic actions. These events are exacerbated in the presence of particular viral infections (HSV-1, influenza virus, and varicella-zoster virus). These pathways share a common microglial-mediated neuroinflammatory route that eventually contributes to the appearance of pathological markers and neurodegeneration typical of the disease. Key: HSV-1 herpes simplex virus -1

There is compelling evidence linking these pathological markers and inflammatory agents/factors. Transgenic mice (TgCRND) treated with an NLRP3 inflammasome inhibitor, JC-124 were shown to decrease microgliosis, Aβ deposits, and oxidative stress (Yin et al. 2018). Another risk factor for inflammation is apolipoprotein Ee4 (APOE e4) (Shi et al. 2017). Interestingly, experiments using animals with a knockdown of the apolipoprotein E gene, show the correct phagocytic/autophagic operation of the microglia/astrocytes to salvage the cells from tau pathology (Wang et al. 2021). The apolipoprotein E gene (Fig. 4) is a risk factor for late-onset AD (Corder et al. 1993). In addition, activation of prostaglandin 2 (which acts as a pro-inflammatory cytokine) is associated with the inhibition of Aβ-related microglial phagocytic activity (Cai et al. 2022), thereby reflecting an inflammatory agent-induced defect in the microglial-autophagy/phagocytic pathway resulting in the aggregation of the misfolded proteins. IL- 6 is a cytokine related to inflammation and infection. It has been shown to build up the hyperphosphorylated tau protein (Quintanilla et al. 2004).

It is pertinent to mention that physiologically, microglia exert a key role in the homeostasis of these proteins by phagocytosis of Aβ and tau proteins (Mandreka-Colucci and Landreth 2010). It clears the soluble form of Aβ via micropinocytosis and fibrillar Aβ peptides by phagocytosis. Thus, microglia serves a neuroprotective role since misfolded Aβ aggregates and tau neurofibrilles are toxic to neurons (Yang et al. 2017). However, as previously stated this protective role may be lost in a chronic state of neuroinflammation, resulting in the reduced clearance and consequent accumulation of these unwanted proteins and thus the manifestation of Aβ plaques and NFTs (Cai et al. 2022). Postmortem studies have shown activated microglia to enclose Aβ deposits and tau-containing dying neuronal cells (Serrano-Pozo et al. 2011). Studies using a transgenic AD mouse model show that tau pathology proceeds through microglial activation via the release of cytokines (Maphis et al. 2015). In addition, the difunctional microglia may contribute to the spread of tau pathology (Ayyubova 2022). Strikingly, there have been conflicting findings from postmortem studies showing that tau pathology is more affiliated with neurodegeneration, loss of neuronal synapses, and cognitive deterioration than β-amyloid-accumulated deposits. In contrast, studies of tau pathology in the mouse model have dispelled any involvement of NFTs with memory loss and neuronal cell destruction (Berger et al. 2007). PET studies have demonstrated an association of the tau NFTs with heightened neuroinflammation in the later prodromal stages of AD (Ismail et al. 2020). This concords with the amyloid cascade hypothesis, which suggests that neuroinflammation mediates the amyloid aggregations during disease progression to prompt tau pathology. Subsequently, the tau aggregates can further exacerbate neuroinflammation and neuronal destruction (Sun et al. 2021). Indeed, experiments using the AD mouse model, show that rutin (a flavonoid glycoside) barred the development of tau aggregation and tau-related cytotoxicity and neuroinflammation (Sun et al. 2021). Some studies have even suggested that the Aβ toxicity is dependent to a certain degree on the presence of NFTs (Roberson et al. 2007).

There has been an association between AD and infections with some pathogens such as influenza virus, **Herpes simplex virus -1 (HSV1**), SARS-CoV-2 virus, HIV, bacteria (Spirobacteria, Streptococcus pneumonia). Around 5.9% of viral encephalitis patients subsequent to a viral infection developed AD (Levine et al. 2023). Furthermore, viral encephalitis increased the risk by 31-fold to develop AD, and are 40 times more likely to have dementia (Levine et al. 2023).

Indeed, viral infections including influenza, and pneumonia exhibited similar associations with dementia (Levine et al 2023). However, others have disagreed with such an association (Imfeld et al. 2016). Nevertheless, the infectious agent may induce systemic and chronic neuroinflammation, which consequently produces cellular/neuronal damage and destruction (Piekut et al. 2022.) In addition, the viral pathogen may also foster typical pathological changes observed in AD, such as Aβ aggregates, and stimulate hyperphosphorylation of tau. The presence of AD pathological markers due to virus/pathogen-related infections endorses the relevance of translating the findings from such studies to the pathogenic processes occurring in AD. A study that employed frequent intermittent lipopolysaccharide-induced infections in mice were reported to impair cognition, disturbed neuronal communications, and had some permanent effects on brain function (Engler-Chiurazzi et al. 2023). Extrapolation of these findings to man would imply that neuroinflammation induced by pathogenic-related infections may have produced a collective destructive effect (s) on the cells. Subsequently, in advancing age, the cells may not be so robust (diminished brain plasticity with age) and they succumb to the onslaught of the cytotoxic processes related to chronic neuroinflammation triggered by the pathogen/virus, leading to cognitive decline amongst other neurodegenerative effects. Indeed, a recent cohort study reported that the influenza vaccination decreases the risk of AD in adults 65 years and above (Bukhbinder et al. 2022).

Similarly, activation of latent HSV1 (present in the brain) has been linked to the manifestation of AD (Mangold and Szpara 2019). Epidemiological studies furnish evidence indicating that frequent HSV1 infection poses a potential risk factor for the illness (Piacentini et al. 2014). Under normal conditions HSV1 resides dormant in the neurons, however, when it is activated it can produce signature pathological changes typical to AD (Cairns et al. 2022). Varicella zoster virus-related infections have been suspected to cause activation of the latent HSV1 virus. In addition, traumatic events such as chemotherapy and radiotherapy can also reactivate HSV1 in the brain (Itzhaki 2016, 2017). The recurrent HSV1 infection probably poses a threat due to neuronal events associated with HSV1 infections such as neuroinflammation, oxidative stress, Aβ accumulation, hyperphosphorylation of tau proteins, and disruption of synaptic cellular communications (Jamieson et al. 1991). These events make up the very foundations for the pathogenesis of AD. Other factors may be involved in viral-specific susceptibility to the illness, such as a genetic component. Indeed, numerous studies have supported the significant risk factor for AD (Fig. 4) in ApoE e4 carriers in association with HSV1 infection (Itzhaki 2017). In contrast, carriers of another isoform, the ApoE e2 allele are protected (Husain et al 2021). Nevertheless, although ApoEε4 is the strongest risk factor for AD, that does not imply that carriers will assuredly develop the disease but they do carry the risk (Yamazaki et al. 2019). Physiologically the isomers of ApoE differentially control the accumulation and clearance of Aβ in the brain and the capacity of binding lipids. However, the presence or expression of ApoE e4 is linked to two mechanisms that can result in neurodegeneration. First, the ApoE e4 may participate in the pathogenesis of AD via Aβ-independent processes, including neuroinflammation, disturbing neuronal-synapsis function, and cholesterol homeostasis. In contrast, the second mechanism is related to the aggregation of Aβ deposits and the cytotoxic events triggered by these deposits (Verghese et al. 2013). Fascinatingly, it has been suggested that the pathogenesis of AD may be linked to mishandling of lipids (Yin 2022). This idea is supported by in vitro studies using human astrocytes, that demonstrated ApoE e4 to disrupt cellular lipid homeostasis (Sienski et al. 2021). Consequently, this can result in cellular destruction, since imbalances in lipids can hinder many vital cellular processes. Therefore, in the diseased state (Fig. 4), the disturbed lipid homeostasis may exert catastrophic effects such as disrupting protein metabolism pathways (which may lead to the build-up of Aβ), amyloidogenesis, disturbing cell signaling, disrupting cellular calcium homeostasis, damaging the blood–brain barrier (allowing easier access to pathogens into the brain) and support the operation of oxidative stress and neuroinflammation (Chew et al. 2020).

### The relevance of Janus-faced molecules in the pathogenesis of PD and AD

There are some molecules/ enzymes/proteins present in the human body that can serve both a physiological and/or a pathological role, thus they are referred to as Janus-faced molecules, in reference to the two-faced Roman god (Sierra et al. 2013). These include: transcription activators, pro-inflammatory cytokines (such as IL-6), nitric oxide, α-synuclein, β-amyloid, and microglial cells.

Inflammatory stimuli including pathogens (such as viruses and bacteria) or non-pathogenic products, or signal molecules (including cytokines, interleukin Il-1β, Il-6) for cellular destruction can interact with toll receptors and receptors (Kaminska et al. 2005). This leads to the activation of these receptors that can trigger a host of important inflammatory pathways including Janus kinase (JAK)-signal transducers, nuclear factor kappa B (NF-κB), mitogen-activated protein kinase (MAPK), and STAT pathways (activator of transcription), finally resulting in the activation of inflammatory cells (Hendrayani et al. 2016; Chen et al. 2017).

**Fig. 5:**
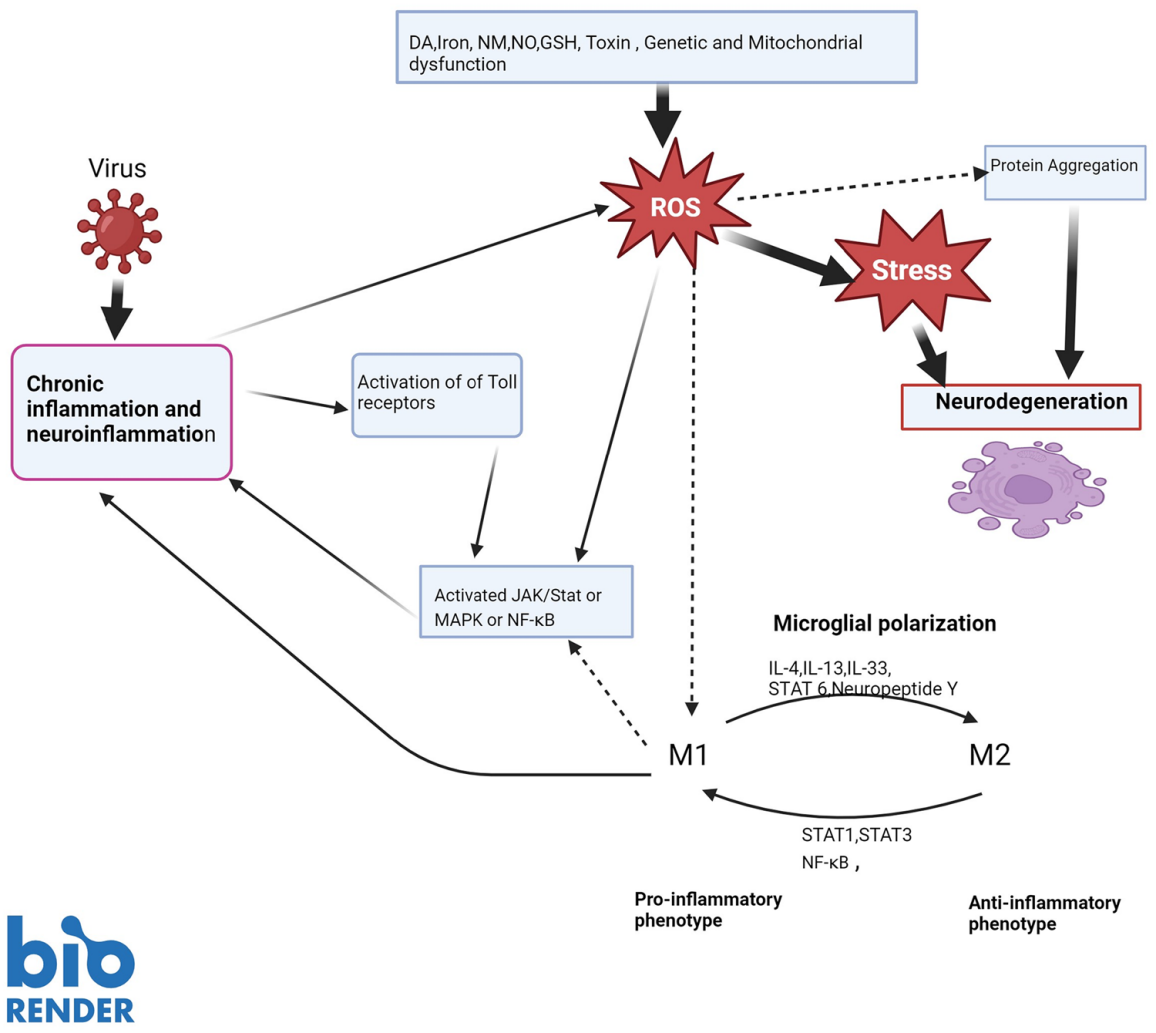
Signal molecules and proteins associated with chronic inflammation and neuroinflammation resulting in neurodegeneration. Molecules released from inflammatory processes can activate Toll-receptors, which in turn stimulate signal molecules (such as JAK/STAT or MAPK or NF-κB) and their pathways. In addition, pro-inflammatory M1 microglia phenotype can also stimulate these signal pathways. The polarization of the M1 microglial is supported by, NF-κB, STAT1, and STAT3, whereas the appearance of anti-inflammatory M2 microglial is favored by IL-4, IL-13, IL-33, STAT 6 and Neuropeptide Y. Consequently, chronic inflammation and neuroinflammation generate reactive oxygen species that induce oxidative stress. Other factors may also initiate the production of reactive oxygen species including dopamine, elevated iron, neuromelanin, nitric oxide, depleted glutathione, endo/exotoxin, genetic predisposition, and mitochondrial dysfunction. Eventually, oxidative stress, accumulation of protein aggregates, and other cytotoxic processes lead to the destruction of cells. Key: ROS reactive oxygen species; JAK/STAT Janus kinase and signal transducer of activation; MAPK mitogen-activated protein kinase; NF-κB nuclear factor kappa B; IL interleukin; DA dopamine; NM neuromelanin; NO nitric oxide; GSH reduced glutathione

The Janus kinase signal transducers (Fig. 5) activate the transcription of the JAK/STAT pathway, which mediates the cellular response to cytokines such as IL-6. JAK-related receptors are activated by ligands via phosphorylation thereby forming docking areas for latent cytoplasmic STAT transcription factors (Harrison 2012). In addition, the binding of the IL-6 cytokines membrane receptors in turn activates the JAK-STAT pathway prompting the transcription of inflammatory genes and activation of cytokines (Boengler et al. 2008). Physiologically the signaling of JAK/STAT is vital for many homeostatic and development processes including immune cell development, and hematopoiesis (Ghoreschi et al. 2009).

However, an imbalance of the JAK-STAT pathway, MAPK, or NF-κB pathways is related to inflammatory, metabolic, and autoimmune diseases (Oekinghaus et al. 2011). This may be ascribed to transcription factor-related cytokine secretion and in addition disturbances on a genetic level since many of the transcription agents regulate inflammatory genes including TNF-α, IL-1, and IL-6, interferons (Rahman and Adcock 2006).

In addition, JAK mutations lead to many diseases such as severe combined immune deficiency, hyper IgE syndromes, and cancer (Jatiani et al. 2010). This highlights the pivotal role of this signaling transducer in the inflammatory response of the immune system, thereby offering a lucrative site for targeting anti-inflammatory therapy.

Interestingly, elevated free radicals (and 8-hydroxy-2-deoxyguanosine) generated from oxidative stress can activate JAK/STAT and other transcription factors, resulting in the enhanced expression of pro-inflammatory cytokines and chemokines. Consequently, this prompts the immune system to respond to inflammatory reactions (Al-Samahari et al. 2015). Extrapolation of these findings to the brain in the diseased state may suggest that perhaps the products of oxidative (and nitrogen) stress such as the free radicals may prompt cytotoxic neuroinflammatory pathways, thereby augmenting the neuronal destruction.

Nitric oxide (NO) is produced by nitric oxide synthase during the conversion of L-arginine to L-citrulline. It plays multiple physiological roles including vasodilation, neurotransmission, regulation of neuronal survival, and activation of the JAK signal transducer. Subsequently, superoxide free radicals (produced by oxidative stress) may react with NO to produce highly oxidant peroxynitrite, which can cause further oxidative cellular damage to DNA, lipids, and proteins (Calabrese et al. 2009; Piacenza et al. 2022). In addition, NO is a potent and highly reactive free radical in its own right, it can thus contribute to the cell carnage associated with neurodegeneration. Neurons appear to be more susceptible to the cytotoxic effects of NO in contrast to astrocytes (Tewari et al. 2020). This is probably due to the presence of a higher content of cellular antioxidant, glutathione in the astrocytes compared to that present in the neurons (Chen et al 2001). This concept is endorsed by the glutathione-dependent protection offered by the astrocytes against oxidative stress.

Neuroinflammation in the brain can lead to excessive production of NO by glia, which can in turn exacerbate cellular deleterious effects associated with chronic neuro-inflammation. Thus, it therefore unequivocally qualifies as a Janus-faced molecule. Indeed, NO can effectively also exert a sinister role in both neurodegeneration and neuroinflammation and is therefore implicated in PD, AD, and other degenerative disorders (Tewari et al. 2020).

Stykel and associates (2022), suggested that about 30% of the proteins present in LB are nitrosylated by reactive nitrogen species. The nitration of α-synuclein by NO may also contribute to its misfolding and aggregation in PD. More importantly, this reflects the involvement of NO and reactive nitrogen species in the degenerative processes occurring in LB-containing diseases or other α-synucleinopathies.

As discussed in depth, α-synuclein also exhibits Janus-faced qualities as demonstrated by its physiological function and pathological role involved in LB formation (Ray et al. 2021).

Interestingly, aggregates of amyloid β proteins are considered to have very harmful properties and exert neurotoxic effects in a myriad of ways in AD as analyzed earlier. However, it has two Janus faces too, since the core hexapeptide structure which is present in many amyloids including Aβ and tau has particularly notable immune suppressant qualities (Steinman et al 2014). Steinman and co-workers (2014) also reported that administration of this hexapeptide (derived from β-amyloid) rapidly decreased levels of pro-inflammatory cytokines (IL-2, IL-6). It would therefore be beneficial in the -management of AD to determine the conditions warranted to activate this component of βamyloid, perhaps to halt or reduce the onslaught of degeneration in AD.

The divergent cellular effects of microglia M1 and M2 clearly exhibit its Janus-faced characteristics. M1 induces inflammatory neurotoxicity, in contrast, M2 exerts anti-inflammatory and neuroprotective actions (Colonna and Butovsky 2017).

It would appear that by virtue of evoking an inflammatory response, viruses may be indirectly involved in the activation of the sinister and cytotoxic component of some of these Janus-faced molecules.

## Conclusion

Neurodegenerative disorders including PD and AD are associated with neuoinflammatory cascades, which may be triggered by eg viral infections. Reviewing these cascades, like ROS/oxidative stress related to dopamine, iron, nitric oxide, loss of glutathione, mitochondrial dysfunction, excitotoxicity, cytotoxic cytokines, microglial M1/M2 activities, JAK-STAT pathway, the role of Toll-receptors, NFκB we aim to elucidate the viral impact leading to accumulation/aggregation of proteins characteristic for PD and AD, α-synuclein (PD), and ß-amyloid respective Tau-protein (AD).

Multiple cytotoxic neuroinflammatory processes leading to protein dyshomeostasis and finally neuronal destruction may depend on “dose” and duration of such pathologic processes induced/triggered by a variety of endotoxins including viruses. As such, involvement of the blood–brain barrier is of crucial importance. The BBB may be intact in the case of neuroinflammation but defect in the case of inflammation. This should be elucidated in future experimental studies and clinical as well as human post mortem brain studies.
